# Data Collection Instruments for Obesogenic Environments in Adults: A Scoping Review

**DOI:** 10.3390/ijerph16081414

**Published:** 2019-04-19

**Authors:** Alba Martínez-García, Eva María Trescastro-López, María Eugenia Galiana-Sánchez, Pamela Pereyra-Zamora

**Affiliations:** Department of Community Nursing, Preventive Medicine and Public Health and History of Science—University of Alicante. Campus de Sant Vicent del Raspeig. Ap. 99, E-03080 Alicante, Spain; galiana@ua.es (M.E.G.-S.); pamela.pereyra@ua.es (P.P.-Z.)

**Keywords:** environment, built environment, food environment, obesity, adult, surveys and questionnaires, measurement

## Abstract

The rise in obesity prevalence has increased research interest in the obesogenic environment and its influence on excess weight. The aim of the present study was to review and map data collection instruments for obesogenic environments in adults in order to provide an overview of the existing evidence and enable comparisons. Through the scoping review method, different databases and webpages were searched between January 1997 and May 2018. Instruments were included if they targeted adults. The documents were categorised as food environment or built environment. In terms of results, 92 instruments were found: 46 instruments measuring the food environment, 42 measuring the built environment, and 4 that characterised both environments. Numerous diverse instruments have been developed to characterise the obesogenic environment, and some of them have been developed based on existing ones; however, most of them have not been validated and there is very little similarity between them, hindering comparison of the results obtained. In addition, most of them were developed and used in the United States and were written in English. In conclusion, there is a need for a robust instrument, improving or combining existing ones, for use within and across countries, and more sophisticated study designs where the environment is contemplated in an interdisciplinary approach.

## 1. Introduction

The rising prevalence of obesity and overweight has generated a growing research interest in determining their causes. One of the key factors that has been identified as contributing to the development of obesity and overweight is the obesogenic environment. This has been defined as “the sum of influences exerted by environments, opportunities and life circumstances that promote obesity in individuals or society” [1]. In other words, the obesogenic environment refers to environmental factors that determine consumption and energy expenditure in individuals and influence the development of obesity. The obesogenic environment is a complex concept, and several models have been developed to conceptualise it and explain how it influences the behaviour of individuals [2,3].

When characterising the obesogenic environment through models, differences have been established between the food environment and the built environment [4]. The food environment is defined as the opportunity to obtain food, which includes the availability, accessibility, advertising, and marketing of food [4]. Food can be accessed in various ways from the food environment: in shops (grocery shops, supermarkets, markets), in catering establishments (bars, restaurants, canteens, takeaway outlets), and in the institutions where people spend part of their day (worksites, schools, homes) [5]. On the other hand, the built environment consists of three elements: physical design, land use (residential, commercial, industrial, and other activities), and the transport system. These make opportunities available for physical activity and for healthy and unhealthy food access. Neighbourhoods providing a range of local facilities within an easy active travel (walking and cycling) distance, with good quality infrastructure (such as well-maintained pavements), and which are regarded as safe and pleasant may support physical activity and influence the propensity of an individual to have an active lifestyle. This environment has generally been studied and characterised by the use of questionnaires and geographic information systems (GIS) [6,7].

Although the concept of obesogenic environment has gained widespread recognition over the last decade [8], studies have used different instruments to identify its components. The developed instruments to measure the obesogenic environment assess either characteristics of it related to the home, worksite, schools, shops, supermarkets, and restaurants, or the possibilities for walking or cycling in a given neighbourhood or city [4,8].

It is often stated that the environment exerts an influence on obesity, but further research is required to identify its specific components and how these influence behaviour in order to be able to modify them. Obesity is the result of multiple and complex factors: to identify all of its causes still remains a research goal. However, studies aimed at characterising the environment have utilized various approaches, methods, metrics, and variables; as a result, it is difficult to compare the evidence and scientific characterisation of the environment, as it continues to be unclear and complex [8]. 

Although the environment impacts all that comes in contact with it, the way in which it influences according to age group is not the same [8]. In addition, there are specific data collection instruments that are classified for each population age group and the characteristics of the studies performed in children are different from those carried out in adults. 

The aim of the present study was to review and map data collection instruments for obesogenic environments in adults in order to provide an overview of the existing evidence and enable comparison. 

## 2. Materials and Methods 

A scoping review was performed since the field is heterogeneous and perhaps not suitable for a more precise systematic review because it is necessary to retrieve information from a variety of documentary sources, including research projects, governmental organisations, and scientific articles indexed in databases. This method is used to facilitate a more exhaustive review of all the literature available on the subject and is useful for answering much broader questions [9]. Moreover, the results of the scoping review will give recommendations for measurements and methods for future research in this field [10].

The scoping review method employed was that proposed by Arksey and O’Malley [11], the Joanna Briggs Institute [12], and PRISMA Extension for Scoping Reviews (PRISMA-ScR) [9], formulating the initial research question: What instruments are available for collecting data on obesogenic environments in adults?

### 2.1. Selection Criteria

Instruments were included if they targeted adults aged 18 to 65 or a mixed-age population sample (adolescents and older adults).

Instruments targeting pregnant women, people with a particular pathology or people living in institutions were excluded, as were population surveys, dietary assessment questionnaires, instruments that were not specifically designed to measure the obesogenic environment, and geographic mapping systems. Instruments unrelated to the subject of the initial research question were also excluded. In the event that the same instrument had been used in more than one study, only the study that described the instrument in most detail was included.

### 2.2. Search Strategy

Different databases were searched, including PubMed, Scopus, PsycInfo, Cochrane, and Web of Science, using the following descriptors: “Surveys and Questionnaires”, “Environment”, “Obesity”, and “Adult”, varying the search strategy according to the database interrogated (Table 1). Controlled language was used to perform the search, using MeSH (Medical Subject Headings) terms (in the case of PubMed), thesauri, and keywords, depending on each database. A search was also performed using free language in the title and abstract fields for all databases, using the term “obesogenic environment”.

In addition, articles cited in previously identified studies that met the inclusion criteria were examined and several webpages was consulted, such as Active Living Research measures, the National Collaborative on Childhood Obesity Research (NCCOR), the National Institutes of Health (NIH), and webpages for existing projects and universities with projects related to the subject and government reports. Documents published worldwide between 1 January 1997 and 31 May 2018 were selected, due to the fact that the concept of “obesogenic environment” appeared for the first time in 1997 [2].

### 2.3. Study Selection

All the identified documents were downloaded in EndNote (EndNote X7 citation management software, Thomson Reuters, Philadelphia, PA, USA). This program was used to remove duplicates and, independently, documents were excluded by title and abstract based on the initial research question as well as inclusion and exclusion criteria. Then, the selected full-text documents were reviewed and those that met the criteria were included.

First, duplicates were eliminated and the documents were assessed by title. The remaining documents were then selected by abstract and full text by two independent researchers. Differences were discussed and resolved by a third reviewer.

### 2.4. Organisation of the Information

Since the obesogenic environment can be measured and classified in different fields, the information was classified according to the data collection instruments and is presented in two tables: the first shows instruments used to analyse the food environment (Table 2), and the second shows those used to analyse the built environment (Table 3). The papers/instruments in Table 2 and Table 3 were ordered by date.

The following information was extracted from each of the documents and entered in a database: author(s)’ names, year of study, name of the instrument, city/country where used, type of instrument, the population/sample targeted, the kind of environment analysed, psychometric properties as instrument validity and instrument reliability according to the author’s criterion, number of items in the instrument, number of different versions, language, and if the instrument had a cultural adaptation and the country where it was culturally adapted and validated. All this information was collected in Table 2 and Table 3. 

The food environment (Table 2) was categorised into six different types: (1) food store (e.g., grocery stores, supermarkets, convenience stores, snack bars, specialty food stores, farmers’ markets, bodegas, and food banks); (2) home food environment (food available at home); (3) macro food environment (e.g., food supply); (4) public facilities to access the food environment (e.g., cafeterias, vending machines or other public locations that offer this type of food); (5) restaurants (including fast food and buffet-type outlets); (6) worksites (cafeterias, vending machines, and snack shops in the workplace) [13,14]; and (7) perceived food environment. 

The built environment (Table 3) was classified according to different aspects of the environment, such as: (1) physical activity environment (places where people are, or can be, physically active); (2) walkability and bikeability in the neighbourhood; (3) worksite physical attributes; (4) neighbourhood design; (5) street-scale features; (6) trail use; and (7) perceived built environment. The places we live, work and so on can either provide or constrain opportunities for physical activity and for healthy and unhealthy food access [8]. 

In the event that an instrument could be used to measure both food and built environments, it was included in both tables (Table 2 and Table 3).

In both tables, types of instruments were classified as checklist or checkbox (a pre-defined list or box of indicator foods which are selected based on predetermined criteria, such as those foods that are identified by the researchers as aligning with current dietary guidance), interview/questionnaire (a pre-determined list of questions that is administered by a trained interviewer or completed by the respondent via self-report), inventory (a form for recording all foods available in a given environment), and market basket (a pre-defined list of foods that represent a range of food choices across a total diet. These foods may be based on foods frequently consumed by the population or may reflect a standardized diet plan), and audit tool (allows systematic observation of the environment, including the presence and qualities of its features) [13,14].

## 3. Results

**Figure 1:**
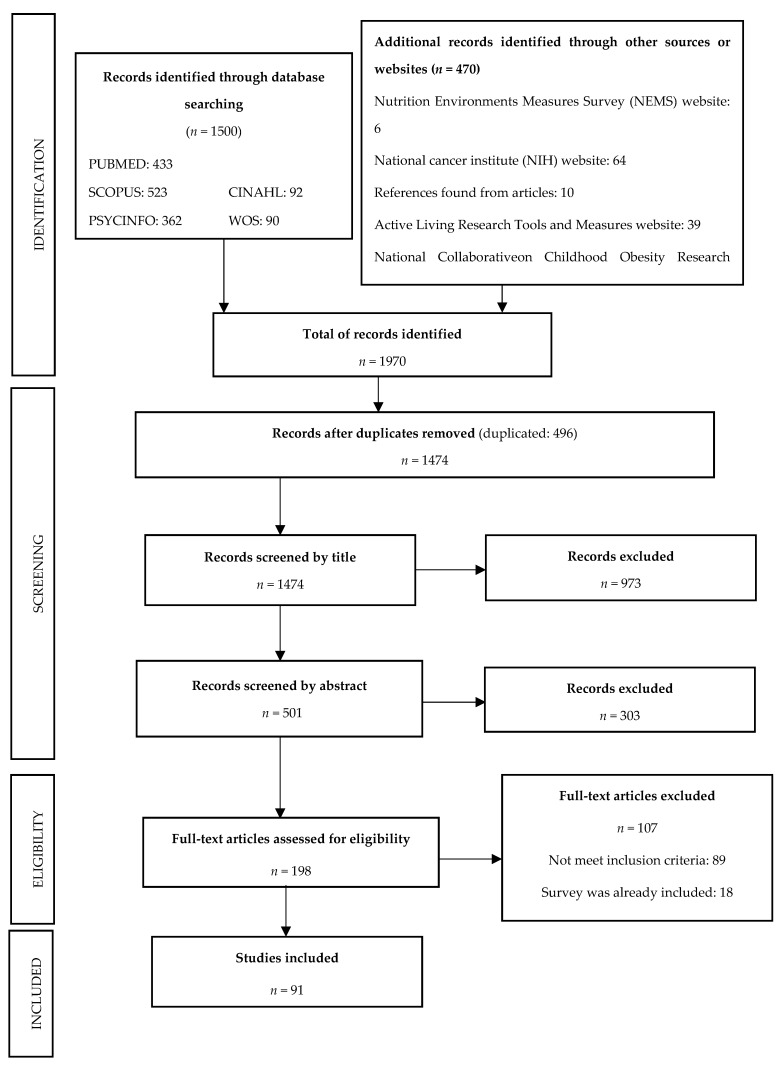
Flow chart illustrating the scoping review study selection process.

Figure 1 shows a flow diagram of the search strategy. As can be seen, 1500 documents were identified, to which 470 obtained from other sources were added, yielding a total of 1970 documents. After deleting duplicates, we obtained 1474 documents for consideration, which were reduced to 198 following an analysis by title and abstract. The full text of these documents was examined, and finally 91 documents were selected due to their characteristics, in which 92 instruments were described (one of the documents reported two instruments).

### 3.1. General Characteristics of the Instruments

A total of 46 instruments were found for characterising the food environment and 42 for analysing the built environment, and 4 instruments that characterised both environments. Of the identified instruments (*n* = 92), 79.4% were developed in the United States, 8.7% in European countries, and 7.6% in Australia, whilst 4.3% were from multiple countries. 

The majority of the studies reviewed have reported psychometric properties (*n* = 64), but out of all the studies (*n* = 92), only 38.0% were reported to be valid and reliable by the authors; 28.3% met some reliability criteria, 3.3% met some criteria of validity, and no mention was made of any criteria in the case of the remaining 30.4%. 

Regarding the types of reliability and validity, studies tended mostly to assess inter-rater reliability (*n* = 34), test–retest reliability (*n* = 30), face-validity (*n* = 12), and construct validity (*n* = 12), whereas only few studies have reported internal-consistency reliability (*n* = 8), criterion-validity (*n* = 8), content-validity (*n* = 5), concurrent-validity (*n* = 4), predictive validity (*n* = 3), and discriminant validity (*n* = 2).

In addition, the instruments used to characterise the built environment were observed to report more psychometric properties than those targeting the food environment, with 88.6% (*n* = 39) using different types of validity and/or reliability, in contrast to the 54.2% (*n* = 26) of the food environment instruments.

With regard to language, almost all instruments were written in English (97.6%), and out of this percentage, only 8.3% were also written in another language (French or Spanish); instruments written in languages other than English (Swedish and Arabic) accounted for only 2.4% of instruments. The most widespread data collection method used in the instruments was the checklist (see Table 2 and Table 3). 

### 3.2. Food Environment

A total of 46 instruments were identified that collected data on the food environment (Table 2). Most of them characterised the food environment of stores (62.5%), followed by restaurants (12.5%), worksites (8.3%), home (8.3%), public facilities (6.3%), perceptions of the food environment (2.2%), and psychosocial factors (2.1%). Only five out of 46 questionnaires considered subjects’ perception of the environment [30,32,46,56,60].

The period 2006 to 2012 witnessed the development of the highest number of instruments. In 2012, two questionnaires were described that characterised the influence of vending machines on the obesogenic environment in public places; one was developed in Australia and the other one in the United States. The instruments contained a median of 33.5 items, with a minimum of 6 and a maximum of 267 items. 

### 3.3. Built Environment

A total of 42 instruments were identified that analysed the built environment (Table 3). Most of them characterised the built environment by studying its physical activity environment (50.0%), followed by neighbourhood design (20.4%), or how these were related to being able to walk or cycle (9.1%). It should be noted that these instruments could collect information on both the population and the built environment (street segments, parks, etc). Only 11 of these 42 instruments considered subjects’ perception of the environment [66,76,78,84,85,91,98,106,108,109,113].

Most of the instruments that collected data on the built environment were developed between 2005 and 2006. However, the most widely used instrument, which has the most adapted and validated versions in different languages and cultures, was developed in 2003 [72]. The median number of items in these instruments was 40, with a minimum of 5 and a maximum of 273 items, showing the high heterogeneity of the number of items of the instruments. However, some instruments have also had short versions developed [65,66,72,114].

### 3.4. Instruments That Characterised Both Environments

Only four instruments were identified that considered both the built and food environments to measure the obesogenic environment. The Worksite Environment Measure (WEM) [26] and the Environment Assessment Tool (EAT) [27] focus only on the worksite environment. The EURO-PREVOB questionnaire [58], and the SPOTLIGHT virtual audit tool (S-VAT) [59] include some types of built and food environments. The S-VAT is particularly focused on collecting information about the food store environment, and the built environment was categorized into walking and cycling, public transport, aesthetics, land use-mix, and physical activity facilities. 

### 3.5. Reference Instruments for the Development of Others

Some of the instruments developed were based on others, such as: [24,26,27,29,34,40,43,46,50,51,54,56,61,63,65,71,76,82,83,84,86,88,90,93,98,99,101,106,107,109,112,113,114]. Table 4 shows the instrument or instruments that were used as a reference to develop others.

## 4. Discussion

The present review has identified the available evidence on the instruments used to characterise the obesogenic environment, in terms of both the food and built environments. This is the first scoping review on this subject, and the first review of instruments that considers both environments in adults. 

Diverse instruments have been developed to characterise the obesogenic environment. Most of them had been developed in the United States and were written in English. Moreover, the majority of the studies reviewed have reported psychometric properties, but out of the all studies only a few were reported to be valid and reliable. 

Some studies conducted in this field until 2015 only consider the influence of the food environment [14,116], or only that of the food store environment [117]. The present review includes, as a novelty, the measures developed in adults both at the food environment and the built environment levels, as well as their types. These can be observed in the tables, which show relevant characteristics to facilitate the selection of one or several instruments for carrying out future research. Additionally, this is the first article where the characteristics of the data collection instruments are classified and described in tables in a clear and fast way for consultation.

The importance of determining the factors that constitute the obesogenic environment has led to the development of a large number of data collection instruments. Most of the identified instruments measured the characteristics of the built or food environments, focusing on one particular area of each environment. These include for example physical activity or the characteristics of an area in the case of the built environment, or food shops, restaurants, the workplace, or home in the case of the food environment. Few instruments considered subjects’ perception of their environment. However, recent research has shown that perception is a mediator between objectively measured exposure and interaction; consequently, studies that combine both are preferable [8].

Although most of the instruments focus only on one type of environment (built or food), there are four that contemplate both. However, they do not meet all the necessary criteria and different types of environments should be contemplated. First of all, WEM [26] and EAT [27] focus on the worksite environment (measuring food and built environments), but only in the workplace. In addition, in the case of EAT, it covers the physical activity environment, and although it includes the food environment, it does so in a lighter way. On the other hand, the questionnaire EURO-PREVOB [58] could be a good instrument to measure obesogenic environments in Europe. Nevertheless, more work is needed to refine and further test the reliability and validity of this instrument in a range of other environments. Although both types of environment are included, they do not include all the types that exist and that need to improve their psychometric properties. Finally, with the S-VAT [59], it does not contemplate all the types of environments that characterize the food and the built environment. Within the food environment it is particularly focused on collecting information about food store environment, and the built environment was categorized into walking and cycling, public transport, aesthetics, land use-mix, and physical activity facilities. For this reason, it cannot be recommended as a robust and reliable tool to assess both environments.

Both the food and built environment data collection instruments showed a wide disparity in the minimum and maximum number of items, which makes comparison difficult. A high number of items reflected inventories or checklists of foods available in shops or restaurants in the case of the food environment, or a list of the characteristics of a neighbourhood or defined area in the case of the built environment.

Although there has been a significant increase over the last decade in the amount of evidence indicating that the environment exerts an influence, there are still unquestionable gaps in current evidence, as shown in this review. Different measures, definitions, and approaches, and continuing attempts to be novel by creating new instruments to measure the environment have merely generated confusion [8].

However, the results showed that the most widely used instruments to characterise the food environment to date are those developed by Glanz et al. (Nutrition Environment Measures Study in Stores (NEMS-S) [22] and Nutrition Environment Measures Study in Restaurants (NEMS-R) [23]) and Oldenburg et al. (Checklist of Health Promotion Environments at Worksites (CHEW) [15]). With regard to the built environment, the most widely used instruments to identify this environment are the Systematic Pedestrian and Cycling Environmental Scan (SPACES) [64], the Neighborhood Environment Walkability Survey (NEWS) [66], the Analitic/Checklist Audit Tool [71], the Active Neighbourhood Checklist [90], and the International Physical Activity Questionnaire (IPAQ) [74], which are often combined with mapping using geographic information systems (GIS), despite limitations since this assumes that food choices are determined primarily by individuals’ proximity to food outlets, without accounting for travel patterns, taste preferences, social norms about where to procure food, or ability to afford foods [13]. They have also served as the basis for various subsequent instruments (Table 4), as well as for studies on the food and built environment.

Few new instruments have been developed for an adult population during the last three years, and this is maybe due to the fact that the majority of studies that were found until May 2018 used the available instruments developed previously, cited in Table 2 and Table 3 [118,119,120,121,122,123,124].

Most of the instruments have been developed and used in the United States. This may be due to concern about the high prevalence of overweight and obesity in this country [125]. Furthermore, there was important role played by the NIH, which has the capacity to finance prevention and intervention initiatives in the development of overweight and obesity, as well as in the development of instruments to characterise the environment [126]. 

Nevertheless, the prevalence of overweight and obesity in Europe is steadily rising and requires more uniform and comprehensive characterisation of the problem and its environments. Although Europe, Australia, and the United States present different food patterns and consumption characteristics, the prevalence of overweight has remained stable or is rising in all three regions [13,125,126,127,128,129]. Influenced by the obesogenic environment, food habits and patterns of consumption are changing in many developed and developing countries, with a marked move towards low vegetable consumption and high animal protein intake [130]. Hence, obesity rates will continue to rise as long as individuals, society, policy-makers, health professionals, social workers, schools, and prevention campaigns continue to give little importance or priority to the obesogenic capacity of environments [131]. Although public health policies have begun to include measures such as banning marketing of unhealthy foods and taxing unhealthy options [132], there is still a need for vigorous action to prevent and reduce obesity by modifying these environments. It is therefore essential for policy-makers to implement effective interventions that tackle the elements involved in the development of obesity, such as certain sectors of the food industry and food marketing and advertising [133]. 

### Limitations

There is no MESH term for the obesogenic environment. This fact hindered the search for evidence and generated more non-meaningful data or information. Database searches would be easier and more accurate if a new term were created referring to this concept and its types (“food environment” and “built environment”). 

## 5. Conclusions

The present study has provided an overview of the instruments used worldwide to measure the obesogenic environment in adults, identifying the components and characteristics of each tool. Numerous diverse instruments have been developed to characterise the obesogenic environment, and some of them have been developed based on existing ones; however, most have not been validated and there is very little similarity between them, hindering comparison of the results obtained. 

Future research should combine validated instruments that characterise the built and food environments and also include subjects’ perception of their environment. In addition, validated tools are required in other countries besides the United States, since those that exist are scarce. In conclusion, there is also a need for robust instruments, improving or combining existing ones, for use within and across countries, and more sophisticated study designs where the environment is contemplated in an interdisciplinary approach. 

## Figures and Tables

**Table 1:** Search strategy.

Database	Search strategy
**PUBMED**	(((obesity[MeSH Terms] OR overweight[MeSH Terms] OR body mass index[MeSH Terms]) AND (environment[MeSH Terms]) AND (adult[MeSH Terms]) AND (surveys and questionnaires[MeSH Terms]))
**SCOPUS**	(TITLE-ABS-KEY (overweight) OR TITLE-ABS-KEY (obesity) OR TITLE-ABS-KEY (body AND mass AND index) AND TITLE-ABS-KEY (environment) AND TITLE-ABS-KEY (adult) AND TITLE-ABS-KEY (surveys AND questionnaires))
**PSYCINFO**	((obesity) OR (overweight) OR (body mass index)) AND (environment) AND (adult) AND (survey AND questionnaires)
**CINAHL**	(AB (overweight or obesity or obese) AND AB adults AND AB environment AND AB (survey or questionnaire or scale or instrument)
**Web Of Science**	TOPIC: (obesity OR overweight) AND TOPIC: (environment) AND TOPIC: (adult) AND TOPIC: (surveys and questionnaires)

**Table 2:** Measures on food environment.

Author (Year)	Instrument	City; Country	Methods	Population/Sample	Environment	Validity	Reliability	Items; Versions	Language	Cultural Adaptation
Oldenburg et al. (2002) [15]	Checklist of Health Promotion Environments at Worksites (CHEW)	Australia	Checklist Interview	Workers	Worksite	Face-validity	Inter-rater	112	English	No
Abarca et al. (2003) [16]	Grocery Store Manager Questions	Arizona; United States/Mexico border communities	Interview/Questionnaire	Grocery store Manager/assistant manager	Food store	No	No	26	English Spanish	No
Sloane et al. (2003) [17]	Healthy Food Assessment Survey	United States	Checklist	African-American community organizations and community residents in the target areas	Food store	No	No	31	English	No
Baker et al. (2006) [18]	Grocery Store Audit Tool, Saint Louis University School of Public Health	Saint Louis; United States	Audit checklist	Community supermarkets	Food store	No	No	92	English	No
Baker et al. (2006) [18]	Fast Food Restaurant Audit Tool, Saint Louis University School of Public Health	Saint Louis; United States	Audit checklist	Community fast food restaurants	Restaurant	No	No	6	English	No
Winkler et al. (2006) [19]	Micro-level Data Collection Worksheet (conventional food store major)	Brisbane City; Australia	Checklist	Trained data collectors recorded detailed information about the availability, variety, and price of 10 fruits and 10 vegetables from nearly all local supermarkets, greengrocers or convenience stores	Food store	No	No	66	English	No
Zenk et al. (2006) [20]	Southwest Chicago Food Store Audit Instrument	Southwest Chicago; United States	Checklist	In-person observations of retail food stores at 2 time points, 2 weeks apart	Food store	No	Test–retest	Food list 16 food items	English	No
Anderson et al. (2007) [21]	Healthy Eating Indicator Shopping Basket (HEISB) Tool	United Kingdom	Checklist	Five contiguous, racially/ethnically and socioeconomically diverse community areas in southwest Chicago	Food store	No	No	35	English	No
Glanz et al. (2007) [22]	Nutrition Environment Measures Study in Stores (NEMS-S)	United States	Interview/Questionnaire	Four neighborhoods that represented four possible combinations of neighbourhood walkability (high/low) and socioeconomic status (high/low) Two other neighborhoods (one high-walkability and one lowwalkability) were selected for pretesting measures	Food store	Face-validity; Construct-validity	Inter-rater and test–retest reliability	93 (11 sections)	English	No
Glanz et al. (2007) [23]	Nutrition Environment Measures Study in Restaurants (NEMS-R)	United States	Interview/Questionnaire	Four neighborhoods were selected to provide diversity in community design (walkable versus nonwalkable) and socioeconomic status (higher and lower income).	Restaurants	Construct validity	Inter-rater and test–retest reliability	25	English	No
Liese et al. (2007) [24]	Food Store Survey	Orangeburg County, South Carolina; United States	Checklist Interview/Questionnaire	Rural county Food stores identified from a database were mapped and presence, location, and store type verified by ground-truthing	Food store	No	Inter-rater reliability	13	English	No
Mujahid et al. (2007) [25]	Neighborhood Health Questionnaire	Baltimore, Maryland; Forsyth County, North Carolina; and New York, New York; United States	Telephone interview/Questionnaire	Residents at three U.S. study sites	Food store Restaurants	No	Internal consistency and test–retest reliability	36	English	No
Shimotsu et al. (2007) [26]	Worksite Environment Measure (WEM) *	United States	Checklist	Two trained raters visited each of the four bus garages and independently completed the survey	Worksite	No	Inter-rater reliability	86 (7 sections)	English	No
DeJoy et al. (2008) [27]	Environmental Assessment Tool (EAT) *	United States	Checklist	Section I completed by site staff and Section II completed by independent observers who toured the site and recorded their observations	Worksite	Concurrent validity; Predictive validity	Inter-rater reliability	105	English	No
Tessier et al. (2008) [28]	“Food supply questionnaire”	Tunis; Africa	Checklist (yes/no)	Food retail outlets	Food store	No	No	146	Arabic	No
Zenk et al. (2008) [29]	Food Environment Audit for Diverse Neighborhoods (FEAD-N)	Detroit; United States	Checklist	Trained observers conducted observations of 167 food stores	Food store	Face-validity; Construct-validity	Inter-rater reliability	267	English	No
Ball et al. (2009) [30]	Food Store Survey	Melbourne; Australia	Checklist	Women aged between 18 and 65 years in each of the 45 neighbourhoods	Food store Perceptions food environment	No	No	53	English	No
Cappelleri et al. (2009) [31]	Power of Food Scale (PFS)	United States	Five-point Likert scale ranging from 1 (do not agree at all) to 5 (strongly agree)	Obese and general population, age not mentioned	Psychological impact of living in food-abundant environments	Content validity	Test–retest reliability; internal consistency	21	English	No
Freedman et al. (2009) [32]	Grocery store survey Perception of Food Environment Scale	Nashville, TN; United States	Checklist	Anyone (adults) shopping at one of the three farmers’ markets that were established at the Boys and Girls Clubs	Food store	No	No	33 8	English	No
French et al. (2009) [33]	Annotated Receipts to Capture Household Purchasing	Minneapolis, Minnesota; United States	Inventory	At least one adult and one child in the household, residence in a private house or apartment within 15 miles of the university, and willingness to be randomized to active intervention or control group	Home food environment	No	No	24	English	No
Fulkerson et al. (2009) [34]	Home Food Inventory (HFI)	Minneapolis, Minnesota; United States	Inventory Yes/no (1/0) response options	Adults and families in which parents completed the HFI	Home food environment	Construct validity; Criterion validity	No	23	English Spanish Somali [31]	No
Song et al. (2009) [35]	Baltimore Healthy Stores Project Survey	Baltimore; United States	Checklist	Low socioeconomic level	Food store	No	No	42	English	No
Nelson MC, Story M (2009) [36]	Dorm room food inventory form	Minnesota; United States	Inventory	Dormitory-residing students from public university	Home food environment	No	No	16	English	No
Minaker et al. (2009) [37]	Assessment tools (food availability and affordability, and establishments)	North America (United States)	Checklist	Food service outlets, (preparing and serving food for immediate consumption), within the geographic boundaries of the campus of the University of Alberta	Restaurants	Face validity; Content validity	No	NM	English	No
Franzen, Smith (2010) [38]	Food Survey Tool for Grocery Stores	Minnesota; United States	Checklist	Grocery stores (13 Hmong/Asian and 2 American)	Food store	No	Test–retest reliability	75	English	No
Futrell et al. (2010) [39]	Food Ubiquity Study	United States	Checklist	Retail stores	Food store	No	No	7	English	No
Gloria et al. (2010) [40]	Texas Nutrition Environment Assessment (TxNEA)	Austin, Texas; United States	Checklist	Convenience stores and grocery stores in one high-income and one low-income neighbourhood	Food store	Face validity	Inter-rater and test–retest reliability	21	English	No
Lucan et al. (2010) [41]	Instrument for Corner Store Snack Food Assessment	Philadelphia; United States	Checklist	Snack foods in 17 Philadelphia corner stores, located in three ethnically distinct, low-income school neighborhoods	Food store	No	No	Depending on the products	English	No
Lake et al. (2010) [42]	Food Environment Classification Tool	Newcastle-Upon-Tyne; United Kingdom	Classification tool	Establishments selling food and/or food products	Food store	NM	Inter-rater	21 points, with 77 sub-categories	English	No
Lee et al. (2010) [43]	Carry-out/fast food restaurant checklist	Baltimore; United States	Checklist	prepared food sources in low-income neighborhoods	Restaurants	No	No	31	English	No
Ghirardelli el at (2011) [44]	Communities of Excellence in Nutrition, Physical Activity, and Obesity Prevention (*CX*^3^) Food Availability and Marketing Survey	United States	Checklist	Twenty six retail food stores in low-income areas	Food store	Face validity	Inter-rater reliability	34	English	No
Gordon et al. (2011) [45]	Retail Food Assessment	New York; United States	Checklist	Low-income and largely Black and Hispanic neighborhoods with high levels of premature morbidity and mortality	Food store	No	No	10	English	No
Gustafson et al. (2011) [46]	Perceived and objective measures of the food store environment	North Carolina; United States	Questionnaire with 5-point Likert Scale (items Adapted from others instruments)	Women aged 40 to 64 years, with incomes at or below 250 % of the federal poverty level, and who had a Body Mass Index between 27.5 and 45.0 kg/m^2^ inclusive	Food store Perceived food environment	No	No	37	English	No
Hosler and Dharssi (2011) [47]	Food Retail Outlet Survey Tool (FROST)	New York; United States	Checklist	39 food stores were visited by the research team	Food store	No	Inter-rater reliability	23	English	No
Stevens et al. (2011) [48]	Exhaustive Home Food Inventory (EHFI)	North Carolina, Durham counties; United States	Inventory	Low-income African-American women with an infant between the ages of 12 and 18 months	Home food environment	No	No	NM	English	No
Suratkar et al. (2011) [49]	Consumer Impact Questionnaire (CIQ)	Baltimore City; United States	Interview	Low-income African-American adult residents	Food store	NM	NM	106	English	No
Ayala et al. (2012) [50]	Grocery Store Observation Guide	South San Diego County; United States	Checklist	Ten stores and 15 supermarkets	Food store	No	Inter-rater reliability	155	English	No
French et al. (2012) [51]	Pharmacy Food Environment: Promoting Sugary Snacks at the Point of Prescription Drug Purchase	Minneapolis; United States	Checklist	Employees from community clinic, hospital and commercial pharmacies	Public facilities	No	Inter-rater reliability	16	English	No
Glanz et al. (2012) [52]	Nutrition Environment Measures Survey-Vending (NEMS-V)	United States	Checklist	Vending machines in Businesses, schools, and communities	Public facilities	NM	Inter-rater reliability Test–retest reliability	Depending on the food	English	No
Kelly et al. (2012) [53]	Measuring Food Environments at Public Transport Sites	Sydney; Australia	Checklist	Vending machines in train stations	Public facilities	No	Inter-rater reliability	8	English	No
Kersten et al. (2012) [54]	Northern CA Retail Food Environment Store Survey	Northern California; United States	Checklist	Small food stores	Food store	No	No	18	English	No
Glanz et al. (2013) [55]	Nutrition Environment Survey for Corner Stores (NEMS-CS)	Philadelphia; United States	Checklist	Corner stores	Food store	NM	Inter-rater reliability Test–retest reliability	111	English	No
Hoehner et al. (2013) [56]	Worksite and Energy Balance Survey (WEBS)	Missouri regions; United States	Interview	Adults 21–65 years old; employed at least 20 hours/week; works at one primary location; primary workplace has ≥5 employees; not pregnant; and no physical limitations to prevent walking or bicycling in the past week	Perceptions food environment worksite	No	Test–retest reliability	84	English	No
Krukowski et al. (2013) [57]	Food Store Selection Questionnaire (FSSQ)	Arkansas communities; United States	Interview	Household food shoppers (93% female, 64% African American), in rural and urban communities	Food store	No	No	49	English	No
Pomerleau et al. (2013) [58]	EURO-PREVOB *	Ankara, Brno, Marseille, Riga, and Sarajevo; Europe	Community Questionnaire	Urban areas	Food and built environment	Content, face and discriminant validity	Inter-rater reliability		English	No
Lakerveld et al. (2014) [59]	SPOTLIGHT Virtual Audit Tool (S-VAT) *	The four largest Dutch cities and their surroundings; west of the Netherlands	Checklist	128 street segments in four Dutch urban neighbourhood that were heterogeneous in socio-economic status and residential density	Food store	Criterion validity	Inter- and intra-observer reliability	40	English	No
Glanz et al. (2015) [60]	Perceived Nutrition Environment Measures Survey (NEMS-P)	Philadelphia; United States	Interview/Questionnaire	Adults (18 or older) residents of higher- and lower-SES neighborhoods	Perceived food environment	Face and content validity	Test–retest reliability	118	English	No
Lo et al. (2015) [61]	NEMS Grab and GO: Food Environment Assessment (NEMS-GG)	Canada; United States	Checklist	Grab-and-go establishments at the University of Toronto	Restaurants: Grab-and-go establishments	Face and construct validity	Inter-rater reliability	7 sections, 22 items	English	No
Ruff et al. (2016) [62]	“A store assessment, a health and behavior survey”	New York City; United States	Interview	Any bodega shopper aged 18+ who purchased food or beverage from a participating store	Food store	No, but included validated questions	No	NM	English	No
DeWeese et al. (2018) [63]	Short-Form Corner Store Audit Tool (SCAT)	New Jersey cities; United States	Checklist	Corner stores	Food store	Criterion validity	Inter-rater reliability	7	English	No

**Table 3:** Measures on built environment.

Author (Year)	Instrument	City; Country	Kind of instrument	Population/Sample	Environment	Validity	Reliability	Items; Versions	Language	Cultural Adaptation
Pikora et al. (2000) [64]	Systematic Pedestrian and Cycling Environmental Scan (SPACES) Instrument	Perth (Western Australia)	Checklist	Sixteen observers with prior experience and trained examined segments within a 400-meter radius of each of the 1803 residences of individuals who had participated in the previous survey of physical activity	Physical activity environment	No	Inter- and intra-rater reliability	37	English	No
Ainsworth et al. (2002) [65]	Environmental Supports for Physical Activity Questionnaire	South Carolina; United States	Multiple choice scale questionnaire- Telephone survey	Adults of geographically selected households	Physical activity environment	Content validity	Test–retest reliability	Original version: 27 Long version: 11 Short version: 5	English	No
Saelens and Sallis, (2002) [66]	Neighborhood Environment Walkability Survey (NEWS) and Neighborhood Environment Walkability Survey–Abbreviated (NEWS-A)	San Diego; United States	Multiple choice scale questionnaire Self-administrated or interview	Adults from two neighborhoods with differing “walkability”, high walkability neighborhood had a mixture of single-family and multiple-family residences, which is consistent with higher residential density, whereas the low-walkability neighbourhood had predominantly single-family homes	Perception of built environment	Construct validity	Test–retest reliability	98 NEWS 54 NEWS-A	English	CNEWS (China) [67] NEWS (Brasil) [68] NEWS (Africa) [69] NEWS (India) [70]
Brownson et al. (2003) [71]	Analytic Audit Tool Checklist Audit Tool	St Louis; United States	“Analytic” (with Likert-scale and ordinal-response choices) “Checklist” (with dichotomous response choices)	Higher income and lower income street segments were audited by different observer pairs	Street-scale environments and rates of physical activity	No	Inter-rater reliability	Analytic: 27 Checklist: 24	English	No
Craig et al. (2003) [72]	International Physical Activity Questionnaire (IPAQ)	12 countries (international)	Checklist	15–64 years	Physical activity environments	Concurrent validity Criterion validity	Test–retest reliability	7 items Short: “Physical activity over the last 7 days” [73]; Long: “Usual physical activity”	English Arabic Croatian Bahasa-Malaysian Danish Dutch (Belgian) Hebrew Greek German French Estonian Korean Icelandic Italian Spanish (Argentina, Columbia, and the United States) Lithuanian Norwegian Persian–Farsi Polish Swedish Taiwanese Vietnamese Turkish	English Arabic Croatian Bahasa-Malaysian Danish Dutch (Belgian) Hebrew Greek German French Estonian Korean Icelandic Italian Spanish (Argentina, Columbia, and the United States) Lithuanian Norwegian Persian–Farsi Polish Swedish Taiwanese Vietnamese Turkish [74]
Emery et al. (2003) [75]	Walking and Bicycling Suitability Assessment (WABSA)	United States	Likert response system Yes/no questions	Two data collectors used walking and bicycling suitability assessment instruments to collect data on 31 road segments	Walkability and bikeability in the neighbourhood	Criterion-related validity	Inter-rater reliability	44: Walking 17 Bicycling 27	English	No
Huston et al. (2003) [76]	NC Six-County Cardiovascular Health (CVH) Survey	Cabarrus, Henderson, Pitt, Robeson, Surry, Wake counties in North Carolina; United States	Cross-sectional telephone survey (mix of surveys)	Age: 18 or more	Perceived built environment	No	No	133	English	No
Clifton et al. (2004) [77]	Pedestrian Environment Data Scan (PEDS) Tool	United States	Audit tool	Segments of a pedestrian network or pathway	Physical activity environment	internal and external validity	Inter- and intra-rater reliability	35	English- Large version (35 items) Spanish- version mini (19 items)	No
Humpel et al. (2004) [78]	Perceptions of Local Environmental Attributes	Australia	Questionnaire	Adults	Physical activity environment	No	Test–Retest Reliability	10	English	No
Rodriguez et al. (2004) [79]	Local Physical Environment	Chapel Hill and Carrboro; United States	Questionnaire	Adults Students and staff commuters to the University of North Carolina in Chapel Hill	Physical activity environment	No	No	NM	English	No
Bedimo-Rung (2005) [80]	Bedimo-Rung Assessment Tools-Direct Observation (BRAT-DO)	New Orleans, Luisiana; United States	Checklist	Fifteen pairs of observers were trained and sent to two parks simultaneously to assess two target areas each	Neighbourhood design Physical characteristics of parks	Criterion validity	Inter-rater reliability	181	English	No
Lee et al. (2005) [81]	Physical Activity Resource Assessment (PARA) instrument	Kansas City, Kansas and Missouri; United States	Check-box	Thirteen urban lower income, high ethnic minority concentration neighborhoods that surrounded public housing developments and four higher income, low ethnic minority concentration comparison neighborhoods	Physical activity environment	No	Test–retest	49	English	No
Armstrong et al. (2006) [82]	Global Physical Activity Questionnaire (GPAQ)	Global Developed by WHO for physical activity surveillance in countries	Questionnaire	Adults	Physical activity environment	Criterion validity	Test–retest reliability	16	English French	No
Boarnet et al. (2006) [83]	Irvine Minnesota Inventory	Southern California and the Minneapolis; United States	Checklist	Street segments	Neighbourhood features and perceived safety	No	Inter-rater reliability	160	English	No
Boehmer et al. (2006) [84]	Telephone Questionnaire Physical Activity and Activity Friendliness of Missouri Ozark Region	Missouri, Tennessee, and Arkansas; United States	Telephone Interview/Questionnaire	18 and older Rural communities	Perceived built environment	No	No	106	English	No
Brownson et al. (2006) [85]	Saint Louis Environment and Physical Activity Instrument	St Louis; United States	Telephone questionnaire	NM	Perceived built environmental	NM	NM	60	English	No
**Giles-Corti B et al.** (2006) [86]	Neighborhood Physical Activity Questionnaire (NPAQ)	Western Australia	Questionnaire	20–71 years (mean 39 years; SD 11.7) A convenience sample of participants drawn from general and academic staff at three universities completed two instruments approximately 1 week apart	Physical Activity Environment	No	Test–retest reliability	28	English	No
Handy et al. (2006) [87]	Perceived Measures of Neighborhood Environment That May Affect Walking	San Francisco Bay area, Silicon Valley Area, Santa Rosa, Sacramento, and Modesto, California; United States	Questionnaire	Adults	Physical Activity Environment	NM	NM	34	English	No
McKenzie et al. (2006) [88]	SOPARC: System for Observing Play and Recreation in Communities	Los Angeles; United States	Check-box	Park and recreation areas, including park users’ physical activity levels, gender, activity modes/types, and estimated age and ethnicity groupings It also collects information on park activity area characteristics (e.g., accessibility, usability, supervision, and organization)	Physical activity environment	Construct validity	Inter-rater	Two boxes	English	No
Troped et al. (2006) [89]	Path Environment Audit Tool (PEAT)	Massachusetts; United States	Audit Tool	Urban, suburban and rural communities	Neighbourhood design	Criterion validity	Inter-observer reliability	36	English	No
Hoehner et al. (2007) [90]	Active Neighborhood Checklist	St. Louis and southeastern Missouri; United States	Checklist- observational tool	Sixty-four street segments in St. Louis and southeastern Missouri were selected among diverse areas that varied with respect to socioeconomic levels, urbanization, and land use	Street-scale features	No	Inter-rater reliability	40	English	No
Shimotsu et al. (2007) [26]	Worksite Environment Measure (WEM) *	United States	Checklist	Two trained raters visited each of the four bus garages and independently completed the survey	Worksite physical activity environment	No	Inter-rater reliability	86 (7 sections)	English	No
DeJoy et al. (2008) [27]	Environmental Assessment Tool (EAT) *	United States	Checklist	Section I completed by site staff and Section II completed by independent observers who toured the site and recorded their observations	Worksite physical activity environment	Concurrent validity; Predictive validity	Inter-rater reliability	105	English	No
Forman et al. (2008) [91]	Perceived Barriers to Walking or Cycling Survey	San Diego (CA); Boston (MA); Cincinnati (OH); United States	Questionnaire Self-administered	Adults parents of children aged 2–18 years	Physical activity environment	Concurrent validity	Test–retest reliability Internal consistency	17	English	No
Ogilvie et al. (2008) [92]	Environmental Characteristics Scale	Glasgow; Scotland	Questionnaire	Adults and those aged 12–18 years Metro/urban population	Physical activity environment	Face validity Concurrent validity	Internal consistency and test–retest reliability	14	English	No
Evenson et al. (2009) [93]	Pregnancy, Infection, and Nutrition (PIN3) Neighborhood Audit Instrument	North Carolina; United States	Audit-instrument. Checklist	Street segments in the research area	Neighbourhood design and walkability	Construct validity	Test–retest reliability	43	English	No
Forsyth et al. (2009) [94]	Twin Cities Walking Survey	Minnesota; United States	Checklist	Adults	Physical activity environment	No	Test–retest reliability	273 (5 sections)	English	No
Purciel et al. (2009) [95]	Measurement Instrument for Urban Design Quantities Related to Walkability	New York; United States	Questionnaire with pictures	Urban design qualities	Built environment and walking behavior	Predictive validity	Inter-rater reliability	25	English	No
Spittaels et al. (2009) [96]	Assessing Levels of PHysical Activity and fitness at population level (ALPHA)	Europe	Questionnaire	General adult populations	Physical activity environment	Predictive validity	Internal consistency Test–retest reliability	49 items grouped in 9 themes	Dutch, English, French, Finnish, German, Spanish	Dutch, English, French, Finnish, German, Spanish
Yousefian et al. (2009) [97]	The Rural Active Living Assessment (RALA) Tools	United States	Checklist	Street segment in seven rural US communities	Neighbourhood design Physical activity environment	No	Inter-rater reliability	81	English	No
Blunt and Hallam (2010) [98]	The Worksite Supportive Environments for Active Living Survey (SEALS)	Kentuky and Mississippi; United States	Self-report questionnaire form using a four-point, Likert-type response scale	20–80 years, and the mean age was 45.5 ± 6.43 years A random sample of regular, full-time employees at two universities	Worksite perceived built environment	face and content validity. Discriminant validity	Internal consistency Construct reliability Test–retest	28	English	No
Kaiser BL et al. (2010) [99]	“Cross-sectional survey”	Wisconsin counties; United States	Questionnaire Five choice-scales Four-point scales	Low-income Anglo and Latino adults Physical activity behaviour and individual, social, and environmental influences on physical activity among adults	Physical activity environment	NM	Test–retest reliability	63	Spanish English	No
Sallis, J.F. et al. (2010) [100]	The Neighborhood Quality of Life Study (NQLS) Survey	32 neighborhoods in Seattle, WA and Baltimore; United States	Checklist	20–65 years residents of neighbourhoods stratified on “walkability” characteristics and median household income	Physical activity environment	NM	NM	222	English	No
Sallis, J.F. et al. (2010) [101]	PANES: Physical Activity Neighborhood Environment Survey	United States	Checklist (self-administered)	Adults recruited from neighborhoods that varied in walkability in three U.S. cities	Walkability and bikeability in the neighbourhood	No	Test–retest reliability	17	English Japanese Italian Nigeria	Nigeria [102] Japanese [103] Italian [104]
Spruijt-Metz D. et al. (2010) [105]	Research on Urban Trail Environments (ROUTES) Trail Use Questionnaire	Chicago, Dallas, and Los Angeles; United States	Checklist Yes/no questions and 9 items with multiple response	STUDY 1: 40 and 60 years of age (10 men and 24 women) Study 2: 490 adults (48% female and 73% white), mean age 48 years	Trail use	Construct validity	Test–retest reliability	43	English	No
Wahlgren L. (2010) [106]	Active Commuting Route Environment Scale (ACRES)	Urban and suburban parts of Greater Stockholm, Sweden; Europe	Questionnaire, 11 or 15-point response scale	20 years or older, living in urban and suburban part of Stockholm County, commute to work or study walking or bicycling at least once a year	Perceived built environment Walkability and bikeability	Criterion-related validity	Test–retest reliability	18	Sweden	No
Kaczynski et al. (2012) [107]	COMMUNITY PARK AUDIT TOOL (CPAT)	Kansas City, Missouri; United States	Checklist	32 adults and 2 teenagers agreed to participate (14 male, 20 female) These included representatives from public health, parks and recreation, planning, nonprofit agencies, youth agencies, education, business associations, municipal legislators, academia, and adult and youth park users and nonusers	Neighbourhood design Physical characteristics of parks	No	Inter-rater reliability	28	English	No
Umstattd et al. (2012) [108]	Development of the Rural Active Living Perceived Environmental Support Scale (RALPESS)	Two rural southeastern states in the United States	Checklist	Adolescents, parents, public school staff, and older adults in two rural southeastern United States counties	Physical activity environment Perceived built environment	Face and content validity	Internal consistency	33	English	No
Adams et al. (2013) [109]	Perceptions of the Environment in the Neighbourhood (PENS)	Cardiff, Kenilworth and Southampton; United Kingdom	Survey scale	Adults living in the study areas	Perceived built environment	No	Test–retest reliability	13	English	No
Duncan et al. (2013) [110]	Office Environment and Sitting Scale (OFFESS)	Australia	Self-administered questionnaire	Adults, workers	Physical Activity Environment Worksite environment	Construct validity	Internal consistency and test re-test reliability	42	English	No
Pomerleau et al. (2013) [58]	EURO-PREVOB *	Ankara, Brno, Marseille, Riga, and Sarajevo; Europe	Community Questionnaire	Urban areas	Food and Built Environment	Content, face and discriminant validity	Inter-rater reliability		English	No
Sasidhara et al. (2014) [111]	SOPARNA: System for Observing Physical Activity and Recreation in Natural Areas	Los Angeles; United States	Checklist—direct observation tool	Wilderness zones and natural open spaces	Physical activity environment in natural zones	Construct validity	Inter-rater	NM	English	No
Malecki et al. (2014) [112]	The Wisconsin Wasabe of the Social and Built Environment (WASABE)	Wisconsin; United States	Multi-dimensional objective audit instrument	Adults aged 21–65 years	Neighbourhood design	Construct validity	Inter-rater reliability	153	English	No
Lakerveld et al. (2014) [59]	SPOTLIGHT virtual audit tool (S-VAT) *	Four largest Dutch cities and their surroundings; west of the Netherlands	Checklist	128 street segments in four Dutch urban neighbourhoods, heterogeneous in socio-economic status and residential density	Neighbourhood design Physical activity environmen Walkability and bikeability in the neighbourhood	Criterion validity	Inter- and intra-observer reliability	40	English	No
Drewnowski et al. (2014) [113]	“20-minute telephone survey” from Seattle Obesity Study (SOS)	King County Washington; United States	Interview/questionnaire Telephone survey	18–65 or older Lower incomen population	Neighbourhood design	NM	NM	22	English	No
Sallis et al. (2015) [114]	Microscale Audit of Pedestrian Streetscapes (MAPS)	United States	Audit Tool	Children, adolescents, younger adults, adults, older adults	Walkability in neighbourhoods	Internal consistency	Inter observer reliability	MAPS-Full: 120 MAPS-Abbreviated: 54 [115] MAPS-Mini: 15	English	No

* Instruments that characterised both environments and are included in Table 2 and Table 3. NM = Not Mentioned

**Table 4:** Reference instruments used as a basis for the development of others.

Reference Instrument (Author; Instrument)	Instrument Developed Based on Reference Instrument (Article Reference Number)
Food environment	
Oldenburg et al.; CHEW [15]	[26,56]
Baker et al.; Grocery Store/Fast Food Restaurant Audit Tool, Saint Louis [16]	[29]
Zenk et al.; Southwest Chicago Food Store Audit Instrument [20]	[29]
Glanz et al.; NEMS-S [22]	[29,40,46]
Glanz et al.; NEMS-R [23]	[43,61]
Glanz et al.; NEMS-CS [55]	[63]
DeJoy et a.l; EAT [27]	[56]
Lake et al.; Food Environment Classification Tool [42]	[105]
Ghirardelli el at; CX^3^ Food Availability and Marketing Survey [44]	[54]
Other tools not included in present review (such as population surveys, etc.)	[24,27,29,34,46,50,51]
Built environment	
Pikora et al.; SPACES [64]	[77,87]
Saelens and Sallis; NEWS [66]	[78,92,97,106]
Brownson et al.; Analitic/Checklist Audit Tool [67]	[77,84,93,104]
Craig et al.; IPAQ [68]	[76,80,95]
Emery et al.; WABSA [69]	[104]
Giles-Corti B et al.; NPAQ [80]	[106]
Hoehner et al.; Active Neighborhood Checklist [84]	[104,105]
Other tools not included in present review (such as population surveys, etc.)	[65,67,70,77,82,87,98,100,104,105,106,107]

## References

[1] Swinburn B., Egger G. (2002). Preventive Strategies against Weight Gain and Obesity. Obes. Rev..

[2] Swinburn B., Egger G., Raza F. (1999). Dissecting Obesogenic Environments: The Development and Application of a Framework for Identifying and Prioritizing Environmental Interventions for Obesity. Prev. Med..

[3] Glanz K., Sallis J.F., Saelens B.E., Frank L.D. (2005). Healthy Nutrition Environments: Concepts and Measures. Am. J. Health Promot..

[4] Lake A., Townshend T. (2006). Obesogenic Environments: Exploring the Built and Food Environments. J. R. Soc. Promot. Health.

[5] Cummins S., Macintyre S. (2006). Food Environments and Obesity—Neighbourhood or Nation?. Int. J. Epidemiol..

[6] Handy S.L., Boarnet M.G., Ewing R., Killingsworth R.E. (2002). How the Built Environment Affects Physical Activity: Views from Urban Planning. Am. J. Prev. Med..

[7] Thornton L.E., Pearce J.R., Kavanagh A.M. (2011). Using Geographic Information Systems (GIS) to Assess the Role of the Built Environment in Influencing Obesity: A Glossary. Int. J. Behav. Nutr. Phys. Act..

[8] Townshend T., Lake A. (2017). Obesogenic Environments: Current Evidence of the Built and Food Environments. Perspect. Public Health.

[9] Tricco A.C., Lillie E., Zarin W., O’Brien K.K., Colquhoun H., Levac D., Moher D., Peters M.D.J., Horsley T., Weeks L. (2018). PRISMA Extension for Scoping Reviews (PRISMA-ScR): Checklist and Explanation. Ann. Intern. Med..

[10] Munn Z., Peters M.D.J., Stern C., Tufanaru C., McArthur A., Aromataris E. (2018). Systematic review or scoping review? Guidance for authors when choosing between a systematic or scoping review approach. BMC Med. Res. Methodol..

[11] Arksey H., O’Malley L. (2005). Scoping Studies: Towards a Methodological Framework. Int. J. Soc. Res. Methodol..

[12] The Joanna Briggs Institute (2015). Joanna Briggs Institute Reviewers’ Manual: Methodology for JBI Scoping Reviews.

[13] National Cancer Institute (NCI). https://www.nih.gov/about-nih/what-we-do/nih-almanac/national-cancer-institute-nci.

[14] Lytle L.A., Sokol R.L. (2017). Measures of the Food Environment: A Systematic Review of the Field, 2007-2015. Health Place.

[15] Oldenburg B., Sallis J.F., Harris D., Owen N. (2002). Checklist of Health Promotion Environments at Worksites (CHEW): Development and Measurement Characteristics. Am. J. Health Promot..

[16] Abarca J., Ramachandran S. (2005). Using Community Indicators to Assess Nutrition in Arizona-Mexico Border Communities. Prev. Chronic Dis..

[17] Sloane D.C., Diamant A.L., Lewis L.B., Yancey A.K., Flynn G., Nascimento L.M., McCarthy W.J., Guinyard J.J., Cousineau M.R. (2003). Improving the Nutritional Resource Environment for Healthy Living Through Community-Based Participatory Research. J. Gen. Intern. Med..

[18] Baker E.A., Schootman M., Barnidge E., Kelly C. (2006). The Role of Race and Poverty in Access to Foods That Enable Individuals to Adhere to Dietary Guidelines. Prev. Chronic Dis..

[19] Winkler E., Turrell G., Patterson C. (2006). Does Living in a Disadvantaged Area Entail Limited Opportunities to Purchase Fresh Fruit and Vegetables in Terms of Price, Availability, and Variety? Findings from the Brisbane Food Study. Health Place.

[20] Zenk S.N., Grigsby-Toussaint D.S., Curry S.J., Berbaum M., Schneider L. (2010). Short-Term Temporal Stability in Observed Retail Food Characteristics. J. Nutr. Educ. Behav..

[21] Anderson A.S., Dewar J., Marshall D., Cummins S., Taylor M., Dawson J., Sparks L. (2007). The Development of a Healthy Eating Indicator Shopping Basket Tool (HEISB) for Use in Food Access Studies—Identification of Key Food Items. Public Health Nutr..

[22] Glanz K., Sallis J.F., Saelens B.E., Frank L.D. (2007). Nutrition Environment Measures Survey in Stores (NEMS-S): Development and Evaluation. Am. J. Prev. Med..

[23] Saelens B.E., Glanz K., Sallis J.F., Frank L.D. (2007). Nutrition Environment Measures Study in Restaurants (NEMS-R): Development and Evaluation. Am. J. Prev. Med..

[24] Liese A.D., Weis K.E., Pluto D., Smith E., Lawson A. (2007). Food Store Types, Availability, and Cost of Foods in a Rural Environment. J. Am. Diet. Assoc..

[25] Mujahid M.S., Diez Roux A.V., Morenoff J.D., Raghunathan T. (2007). Assessing the Measurement Properties of Neighborhood Scales: From Psychometrics to Ecometrics. Am. J. Epidemiol..

[26] Shimotsu S.T., French S.A., Gerlach A.F., Hannan P.J. (2007). Worksite Environment Physical Activity and Healthy Food Choices: Measurement of the Worksite Food and Physical Activity Environment at Four Metropolitan Bus Garages. Int. J. Behav. Nutr. Phys. Act..

[27] DeJoy D.M., Wilson M.G., Goetzel R.Z., Ozminkowski R.J., Wang S., Baker K.M., Bowen H.M., Tully K.J. (2008). Development of the Environmental Assessment Tool (EAT) to Measure Organizational Physical and Social Support for Worksite Obesity Prevention Programs. J. Occup. Environ. Med..

[28] Tessier S., Traissac P., Maire B., Bricas N., Eymard-Duvernay S., El Ati J., Delpeuch F. (2008). Regular Users of Supermarkets in Greater Tunis Have a Slightly Improved Diet Quality. J. Nutr..

[29] Izumi B.T., Zenk S.N., Schulz A.J., Mentz G.B., Sand S.L., de Majo R.F., Wilson C., Odoms-Young A. (2012). Inter-Rater Reliability of the Food Environment Audit for Diverse Neighborhoods (FEAD-N). J. Urban Health.

[30] Ball K., Timperio A., Crawford D. (2009). Neighbourhood Socioeconomic Inequalities in Food Access and Affordability. Health Place.

[31] Cappelleri J.C., Bushmakin A.G., Gerber R.A., Leidy N.K., Sexton C.C., Karlsson J., Lowe M.R. (2009). Evaluating the Power of Food Scale in Obese Subjects and a General Sample of Individuals: Development and Measurement Properties. Int. J. Obes..

[32] Freedman D.A. (2009). Local Food Environments: They’re All Stocked Differently. Am. J. Community Psychol..

[33] French S.A., Wall M., Mitchell N.R., Shimotsu S.T., Welsh E. (2009). Annotated Receipts Capture Household Food Purchases from a Broad Range of Sources. Int. J. Behav. Nutr. Phys. Act..

[34] Fulkerson J.A., Nelson M.C., Lytle L., Moe S., Heitzler C., Pasch K.E. (2008). The Validation of a Home Food Inventory. Int. J. Behav. Nutr. Phys. Act..

[35] Song H.-J., Gittelsohn J., Kim M., Suratkar S., Sharma S., Anliker J. (2009). A Corner Store Intervention in a Low-Income Urban Community Is Associated with Increased Availability and Sales of Some Healthy Foods. Public Health Nutr..

[36] Nelson M.C., Story M. (2009). Food Environments in University Dorms: 20,000 Calories per Dorm Room and Counting. Am. J. Prev. Med..

[37] Minaker L.M., Raine K.D., Cash S.B. (2009). Measuring the Food Service Environment: Development and Implementation of Assessment Tools. Can. J. Public Health.

[38] Franzen L., Smith C. (2010). Food System Access, Shopping Behavior, and Influences on Purchasing Groceries in Adult Hmong Living in Minnesota. Am. J. Health Promot..

[39] Farley T.A., Baker E.T., Futrell L., Rice J.C. (2010). The Ubiquity of Energy-Dense Snack Foods: A National Multicity Study. Am. J. Public Health.

[40] Gloria C.T., Steinhardt M.A. (2010). Texas Nutrition Environment Assessment of Retail Food Stores (TxNEA-S): Development and Evaluation. Public Health Nutr..

[41] Lucan S.C., Karpyn A., Sherman S. (2010). Storing Empty Calories and Chronic Disease Risk: Snack-Food Products, Nutritive Content, and Manufacturers in Philadelphia Corner Stores. J. Urban Health.

[42] Lake A.A., Burgoine T., Greenhalgh F., Stamp E., Tyrrell R. (2010). The Foodscape: Classification and Field Validation of Secondary Data Sources. Health Place.

[43] Lee S.H., Rowan M.T., Powell L.M., Newman S., Klassen A.C., Frick K.D., Anderson J., Gittelsohn J. (2010). Characteristics of Prepared Food Sources in Low-Income Neighborhoods of Baltimore City. Ecol. Food Nutr..

[44] Ghirardelli A., Quinn V., Sugerman S. (2011). Reliability of a Retail Food Store Survey and Development of an Accompanying Retail Scoring System to Communicate Survey Findings and Identify Vendors for Healthful Food and Marketing Initiatives. J. Nutr. Educ. Behav..

[45] Gordon C., Purciel-Hill M., Ghai N.R., Kaufman L., Graham R., Van Wye G. (2011). Measuring Food Deserts in New York City’s Low-Income Neighborhoods. Health Place.

[46] Gustafson A.A., Sharkey J., Samuel-Hodge C.D., Jones-Smith J., Folds M.C., Cai J., Ammerman A.S. (2011). Perceived and Objective Measures of the Food Store Environment and the Association with Weight and Diet among Low-Income Women in North Carolina. Public Health Nutr..

[47] Hosler A.S., Dharssi A. (2011). Reliability of a Survey Tool for Measuring Consumer Nutrition Environment in Urban Food Stores. J. Public Health Manag. Pract..

[48] Stevens J., Bryant M., Wang L., Borja J., Bentley M.E. (2011). Exhaustive Measurement of Food Items in the Home Using a Universal Product Code Scanner. Public Health Nutr..

[49] Suratkar S., Gittelsohn J., Song H.-J., Anliker J.A., Sharma S., Mattingly M. (2010). Food Insecurity Is Associated with Food-Related Psychosocial Factors and Behaviors Among Low-Income African American Adults in Baltimore City. J. Hunger Environ. Nutr..

[50] Emond J.A., Madanat H.N., Ayala G.X. (2012). Do Latino and Non-Latino Grocery Stores Differ in the Availability and Affordability of Healthy Food Items in a Low-Income, Metropolitan Region?. Public Health Nutr..

[51] Whitehouse A., Simon A., French S.A., Wolfson J. (2012). Availability of Snacks, Candy and Beverages in Hospital, Community Clinic and Commercial Pharmacies. Public Health Nutr..

[52] Voss C., Klein S., Glanz K., Clawson M. (2012). Nutrition Environment Measures Survey-Vending: Development, Dissemination, and Reliability. Health Promot. Pract..

[53] Kelly B., Flood V.M., Bicego C., Yeatman H. (2012). Derailing Healthy Choices: An Audit of Vending Machines at Train Stations in NSW. Health Promot. J. Aust..

[54] Kersten E., Laraia B., Kelly M., Adler N., Yen I.H. (2012). Small Food Stores and Availability of Nutritious Foods: A Comparison of Database and in-Store Measures, Northern California, 2009. Prev. Chronic Dis..

[55] Cavanaugh E., Mallya G., Brensinger C., Tierney A., Glanz K. (2013). Nutrition Environments in Corner Stores in Philadelphia. Prev. Med..

[56] Hoehner C.M., Budd E.L., Marx C.M., Dodson E.A., Brownson R.C. (2013). Development and Reliability Testing of the Worksite and Energy Balance Survey. J. Public Health Manag. Pract..

[57] Krukowski R.A., Sparks C., DiCarlo M., McSweeney J., West D.S. (2013). There’s More to Food Store Choice than Proximity: A Questionnaire Development Study. BMC Public Health.

[58] Pomerleau J., Knai C., Foster C., Rutter H., Darmon N., Derflerova Brazdova Z., Hadziomeragic A.F., Pekcan G., Pudule I., Robertson A. (2013). Measuring the Food and Built Environments in Urban Centres: Reliability and Validity of the EURO-PREVOB Community Questionnaire. Public Health.

[59] Bethlehem J.R., Mackenbach J.D., Ben-Rebah M., Compernolle S., Glonti K., Bárdos H., Rutter H.R., Charreire H., Oppert J.-M., Brug J. (2014). The SPOTLIGHT Virtual Audit Tool: A Valid and Reliable Tool to Assess Obesogenic Characteristics of the Built Environment. Int. J. Health Geogr..

[60] Green S.H., Glanz K. (2015). Development of the Perceived Nutrition Environment Measures Survey. Am. J. Prev. Med..

[61] Lo B.K.C., Minaker L., Chan A.N.T., Hrgetic J., Mah C.L. (2016). Adaptation and Validation of a Nutrition Environment Measures Survey for University Grab-and-Go Establishments. Can. J. Diet. Pract. Res..

[62] Ruff R.R., Akhund A., Adjoian T. (2016). Small Convenience Stores and the Local Food Environment: An Analysis of Resident Shopping Behavior Using Multilevel Modeling. Am. J. Health Promot..

[63] DeWeese R.S., Todd M., Karpyn A., Yedidia M.J., Kennedy M., Bruening M., Wharton C.M., Ohri-Vachaspati P. (2018). Short-Form Audit Instrument for Assessing Corner Store Healthfulness. Am. J. Health Promot..

[64] Pikora T.J., Bull F.C.L., Jamrozik K., Knuiman M., Giles-Corti B., Donovan R.J. (2002). Developing a Reliable Audit Instrument to Measure the Physical Environment for Physical Activity. Am. J. Prev. Med..

[65] Kirtland K.A., Porter D.E., Addy C.L., Neet M.J., Williams J.E., Sharpe P.A., Neff L.J., Kimsey C.D., Ainsworth B.E. (2003). Environmental Measures of Physical Activity Supports: Perception versus Reality. Am. J. Prev. Med..

[66] Saelens B.E., Sallis J.F., Black J.B., Chen D. (2003). Neighborhood-Based Differences in Physical Activity: An Environment Scale Evaluation. Am. J. Public Health.

[67] (2011). Evaluation on reliability and validity of Chinese Walkable Environment Scale for urban community residents. Chin. J. Public Health.

[68] Malavasi L.M., Duarte M.F.S., Both J., Reis R.S. (2007). Escala de mobilidade ativa no ambiente comunitário - NEWS Brasil: retradução e reprodutibilidade. Rev. Bras. Cineantropom Desempenho Hum..

[69] Oyeyemi A.L., Conway T.L., Adedoyin R.A., Akinroye K.K., Aryeetey R., Assah F., Cain K.L., Gavand K.A., Kasoma S.S., Kolbe-Alexander T.L. (2017). Construct Validity of the Neighborhood Environment Walkability Scale for Africa. Med. Sci. Sports Exerc..

[70] Adlakha D., Hipp J.A., Brownson R.C. (2016). Adaptation and Evaluation of the Neighborhood Environment Walkability Scale in India (NEWS-India). Int. J. Environ. Res. Public Health.

[71] Reliability of Two Instruments for Auditing the Environment for Physical Activity | Active Living Research. https://activelivingresearch.org/reliability-two-instruments-auditing-environment-physical-activity.

[72] Craig C.L., Marshall A.L., Sjöström M., Bauman A.E., Booth M.L., Ainsworth B.E., Pratt M., Ekelund U., Yngve A., Sallis J.F. (2003). International Physical Activity Questionnaire: 12-Country Reliability and Validity. Med. Sci. Sports Exerc..

[73] Lee P.H., Macfarlane D.J., Lam T., Stewart S.M. (2011). Validity of the International Physical Activity Questionnaire Short Form (IPAQ-SF): A Systematic Review. Int. J. Behav. Nutr. Phys. Act..

[74] International Physical Activity Questionnaire. https://sites.google.com/site/theipaq/.

[75] Emery J., Crump C., Bors P. (2003). Reliability and Validity of Two Instruments Designed to Assess the Walking and Bicycling Suitability of Sidewalks and Roads. Am. J. Health Promot..

[76] Huston S.L., Evenson K.R., Bors P., Gizlice Z. (2003). Neighborhood Environment, Access to Places for Activity, and Leisure-Time Physical Activity in a Diverse North Carolina Population. Am. J. Health Promot..

[77] Clifton K.J., Livi Smith A.D., Rodriguez D. (2007). The Development and Testing of an Audit for the Pedestrian Environment. Landsc. Urban Plan..

[78] Humpel N., Marshall A.L., Leslie E., Bauman A., Owen N. (2004). Changes in Neighborhood Walking Are Related to Changes in Perceptions of Environmental Attributes. Ann. Behav. Med..

[79] Rodrĺguez D.A., Joo J. (2004). The Relationship between Non-Motorized Mode Choice and the Local Physical Environment. Transp. Res. Part D Transp. Environ..

[80] Bedimo-Rung A.L., Gustat J., Tompkins B.J., Rice J., Thomson J. (2006). Development of a Direct Observation Instrument to Measure Environmental Characteristics of Parks for Physical Activity. J. Phys. Act Health.

[81] Lee R.E., Booth K.M., Reese-Smith J.Y., Regan G., Howard H.H. (2005). The Physical Activity Resource Assessment (PARA) Instrument: Evaluating Features, Amenities and Incivilities of Physical Activity Resources in Urban Neighborhoods. Int. J. Behav. Nutr. Phys. Act..

[82] Armstrong T., Bull F. (2006). Development of the World Health Organization Global Physical Activity Questionnaire (GPAQ). J. Public Health.

[83] Boarnet M.G., Day K., Alfonzo M., Forsyth A., Oakes M. (2006). The Irvine-Minnesota Inventory to Measure Built Environments: Reliability Tests. Am. J. Prev. Med..

[84] Boehmer T.K., Lovegreen S.L., Haire-Joshu D., Brownson R.C. (2006). What Constitutes an Obesogenic Environment in Rural Communities?. Am. J. Health Promot..

[85] Brownson R.C., Chang J.J., Eyler A.A., Ainsworth B.E., Kirtland K.A., Saelens B.E., Sallis J.F. (2004). Measuring the Environment for Friendliness toward Physical Activity: A Comparison of the Reliability of 3 Questionnaires. Am. J. Public Health.

[86] Giles-Corti B., Timperio A., Cutt H., Pikora T.J., Bull F.C.L., Knuiman M., Bulsara M., Van Niel K., Shilton T. (2006). Development of a Reliable Measure of Walking within and Outside the Local Neighborhood: RESIDE’s Neighborhood Physical Activity Questionnaire. Prev. Med..

[87] Handy S., Cao X., Mokhtarian P.L. (2006). Self-Selection in the Relationship between the Built Environment and Walking: Empirical Evidence from Northern California. J. Am. Plan. Assoc..

[88] McKenzie T.L., Cohen D.A., Sehgal A., Williamson S., Golinelli D. (2006). System for Observing Play and Recreation in Communities (SOPARC): Reliability and Feasibility Measures. J. Phys. Act. Health.

[89] Troped P.J., Cromley E.K., Fragala M.S., Melly S.J., Hasbrouck H.H., Gortmaker S.L., Brownson R.C. (2006). Development and Reliability and Validity Testing of an Audit Tool for Trail/Path Characteristics: The Path Environment Audit Tool (PEAT). J. Phys. Act. Health.

[90] Hoehner C.M., Ivy A., Ramirez L.K.B., Handy S., Brownson R.C. (2007). Active Neighborhood Checklist: A User-Friendly and Reliable Tool for Assessing Activity Friendliness. Am. J. Health Promot..

[91] Forman H., Kerr J., Norman G.J., Saelens B.E., Durant N.H., Harris S.K., Sallis J.F. (2008). Reliability and Validity of Destination-Specific Barriers to Walking and Cycling for Youth. Prev. Med..

[92] Ogilvie D., Mitchell R., Mutrie N., Petticrew M., Platt S. (2008). Perceived Characteristics of the Environment Associated with Active Travel: Development and Testing of a New Scale. Int. J. Behav. Nutr. Phys. Act..

[93] Evenson K.R., Sotres-Alvarez D., Herring A.H., Messer L., Laraia B.A., Rodríguez D.A. (2009). Assessing Urban and Rural Neighborhood Characteristics Using Audit and GIS Data: Derivation and Reliability of Constructs. Int. J. Behav. Nutr. Phys. Act..

[94] Forsyth A., Oakes J.M., Schmitz K.H. (2009). Test–retest Reliability of the Twin Cities Walking Survey. J. Phys. Act. Health.

[95] Purciel M., Neckerman K.M., Lovasi G.S., Quinn J.W., Weiss C., Bader M.D.M., Ewing R., Rundle A. (2009). Creating and Validating GIS Measures of Urban Design for Health Research. J. Environ. Psychol..

[96] Spittaels H., Verloigne M., Gidlow C., Gloanec J., Titze S., Foster C., Oppert J.-M., Rutter H., Oja P., Sjöström M. (2010). Measuring Physical Activity-Related Environmental Factors: Reliability and Predictive Validity of the European Environmental Questionnaire ALPHA. Int. J. Behav. Nutr. Phys. Act..

[97] Yousefian A., Hennessy E., Umstattd M.R., Economos C.D., Hallam J.S., Hyatt R.R., Hartley D. (2010). Development of the Rural Active Living Assessment Tools: Measuring Rural Environments. Prev. Med..

[98] Blunt G.H., Hallam J.S. (2010). The Worksite Supportive Environments for Active Living Survey: Development and Psychometric Properties. Am. J. Health Promot..

[99] Kaiser B.L., Brown R.L., Baumann L.C. (2010). Perceived Influences on Physical Activity and Diet in Low-Income Adults from Two Rural Counties. Nurs. Res..

[100] Frank L.D., Sallis J.F., Saelens B.E., Leary L., Cain K., Conway T.L., Hess P.M. (2010). The Development of a Walkability Index: Application to the Neighborhood Quality of Life Study. Br. J. Sports Med..

[101] Sallis J.F., Kerr J., Carlson J.A., Norman G.J., Saelens B.E., Durant N., Ainsworth B.E. (2010). Evaluating a Brief Self-Report Measure of Neighborhood Environments for Physical Activity Research and Surveillance: Physical Activity Neighborhood Environment Scale (PANES). J. Phys. Act. Health.

[102] Oyeyemi A.L., Sallis J.F., Oyeyemi A.Y., Amin M.M., De Bourdeaudhuij I., Deforche B. (2013). Adaptation, Test–retest Reliability, and Construct Validity of the Physical Activity Neighborhood Environment Scale in Nigeria (PANES-N). J. Phys. Act. Health.

[103] (2018). International physical activity questionnaire. https://sites.google.com/site/theipaq/references.

[104] Sallis J.F. Physical Activity Neighborhood Environment Survey (PANES). http://sallis.ucsd.edu/measure_panes.html.

[105] Spruijt-Metz D., Wolch J., Jerrett M., Byrne J., Hsieh S., Myles R., Xie B., Wang L., Chou C.-P., Reynolds K.D. (2010). Development, Reliability, and Validity of an Urban Trail Use Survey. Am. J. Health Promot..

[106] Wahlgren L., Schantz P. (2011). Bikeability and Methodological Issues Using the Active Commuting Route Environment Scale (ACRES) in a Metropolitan Setting. BMC Med. Res. Methodol..

[107] Kaczynski A.T., Wilhelm Stanis S.A., Besenyi G.M. (2012). Development and Testing of a Community Stakeholder Park Audit Tool. Am. J. Prev. Med..

[108] Umstattd M.R., Baller S.L., Hennessy E., Hartley D., Economos C.D., Hyatt R.R., Yousefian A., Hallam J.S. (2012). Development of the Rural Active Living Perceived Environmental Support Scale (RALPESS). J. Phys. Act. Health.

[109] Adams E.J., Goodman A., Sahlqvist S., Bull F.C., Ogilvie D. (2013). Correlates of Walking and Cycling for Transport and Recreation: Factor Structure, Reliability and Behavioural Associations of the Perceptions of the Environment in the Neighbourhood Scale (PENS). Int. J. Behav. Nutr. Phys. Act..

[110] Duncan M.J., Rashid M., Vandelanotte C., Cutumisu N., Plotnikoff R.C. (2013). Development and Reliability Testing of a Self-Report Instrument to Measure the Office Layout as a Correlate of Occupational Sitting. Int. J. Behav. Nutr. Phys. Act..

[111] SOPARNA: System for Observing Physical Activity and Recreation in Natural Areas|Active Living Research. https://activelivingresearch.org/soparna-system-observing-physical-activity-and-recreation-natural-areas.

[112] Malecki K.C., Engelman C.D., Peppard P.E., Nieto F.J., Grabow M.L., Bernardinello M., Bailey E., Bersch A.J., Walsh M.C., Lo J.Y. (2014). The Wisconsin Assessment of the Social and Built Environment (WASABE): A Multi-Dimensional Objective Audit Instrument for Examining Neighborhood Effects on Health. BMC Public Health.

[113] Drewnowski A., Aggarwal A., Rehm C.D., Cohen-Cline H., Hurvitz P.M., Moudon A.V. (2014). Environments Perceived as Obesogenic Have Lower Residential Property Values. Am. J. Prev. Med..

[114] Sallis J.F. (2015). Is Your Neighborhood Designed to Support Physical Activity? A Brief Streetscape Audit Tool. Prev. Chronic Dis..

[115] Cain K.L., Gavand K.A., Conway T.L., Geremia C.M., Millstein R.A., Frank L.D., Saelens B.E., Adams M.A., Glanz K., King A.C. (2017). Developing and Validating an Abbreviated Version of the Microscale Audit for Pedestrian Streetscapes (MAPS-Abbreviated). J. Transp. Health.

[116] McKinnon R.A., Reedy J., Morrissette M.A., Lytle L.A., Yaroch A.L. (2009). Measures of the Food Environment: A Compilation of the Literature, 1990–2007. Am. J. Prev. Med..

[117] Glanz K., Johnson L., Yaroch A.L., Phillips M., Ayala G.X., Davis E.L. (2016). Measures of Retail Food Store Environments and Sales: Review and Implications for Healthy Eating Initiatives. J. Nutr. Educ. Behav..

[118] Mackenbach J.D., Lakerveld J., van Lenthe F.J., Bárdos H., Glonti K., Compernolle S., De Bourdeaudhuij I., Oppert J.-M., Roda C., Rutter H. (2016). Exploring Why Residents of Socioeconomically Deprived Neighbourhoods Have Less Favourable Perceptions of Their Neighbourhood Environment than Residents of Wealthy Neighbourhoods. Obes. Rev..

[119] Shaver E.R., Sadler R.C., Hill A.B., Bell K., Ray M., Choy-Shin J., Lerner J., Soldner T., Jones A.D. (2018). The Flint Food Store Survey: Combining Spatial Analysis with a Modified Nutrition Environment Measures Survey in Stores (NEMS-S) to Measure the Community and Consumer Nutrition Environments. Public Health Nutr..

[120] Camden A., Levy J., Bassil K., Vanderlinden L., Barnett O.W., Minaker L.M., Mulligan K., Campbell M. (2018). A Census of Midsize to Large Supermarkets in Toronto: A Cross-Sectional Analysis of the Consumer Nutrition Environment. J. Nutr. Educ. Behav..

[121] Lindberg R., Sidebottom A.C., McCool B., Pereira R.F., Sillah A., Boucher J.L. (2018). Changing the Restaurant Food Environment to Improve Cardiovascular Health in a Rural Community: Implementation and Evaluation of the Heart of New Ulm Restaurant Programme. Public Health Nutr..

[122] Díez J., Valiente R., Ramos C., García R., Gittelsohn J., Franco M. (2017). The Mismatch between Observational Measures and Residents’ Perspectives on the Retail Food Environment: A Mixed-Methods Approach in the Heart Healthy Hoods Study. Public Health Nutr..

[123] Hearst M.O., Fulkerson J.A., Parke M., Martin L. (2013). Validation of a Home Food Inventory among Low-Income Spanish- and Somali-Speaking Families. Public Health Nutr..

[124] Jilcott Pitts S.B., Wu Q., Truesdale K.P., Laska M.N., Grinchak T., McGuirt J.T., Haynes-Maslow L., Bell R.A., Ammerman A.S. (2017). Baseline Assessment of a Healthy Corner Store Initiative: Associations between Food Store Environments, Shopping Patterns, Customer Purchases, and Dietary Intake in Eastern North Carolina. Int. J. Environ. Res. Public Health.

[125] Global Obesity Observatory|Home. https://www.worldobesitydata.org/map/overview-adults.

[126] National Institutes of Health (NIH). https://www.nih.gov/.

[127] Overweight and obesity—BMI statistics—Statistics Explained. https://ec.europa.eu/eurostat/statistics-explained/index.php/Overweight_and_obesity_-_BMI_statistics.

[128] Overweight & obesity Overview. https://www.aihw.gov.au/reports-data/behaviours-risk-factors/overweight-obesity/overview.

[129] Congdon P. (2017). Variations in Obesity Rates between US Counties: Impacts of Activity Access, Food Environments, and Settlement Patterns. Int. J. Environ. Res. Public Health.

[130] Poli A. (2010). The Food Pyramid and the Environmental Pyramid. Barilla Center for Food & Nutrition. http://www.fao.org/ag/humannutrition.

[131] Roda C., Charreire H., Feuillet T., Mackenbach J.D., Compernolle S., Glonti K., Ben Rebah M., Bárdos H., Rutter H., McKee M. (2016). Mismatch between Perceived and Objectively Measured Environmental Obesogenic Features in European Neighbourhoods. Obes. Rev..

[132] WHO Regional Office for Europe (2014). Prevention and Control of Noncommunicable Diseases in the European Region: A Progress Report.

[133] Visscher T.L.S., Lakerveld J., Olsen N., Küpers L., Ramalho S., Keaver L., Brei C., Bjune J.-I., Ezquerro S., Yumuk V. (2017). Perceived Health Status: Is Obesity Perceived as a Risk Factor and Disease?. Obes. Facts.

